# Molecular epidemiology of mosquitoes for the transmission of forest malaria in south-central Vietnam

**DOI:** 10.1186/s41182-017-0065-6

**Published:** 2017-10-12

**Authors:** Yoshimasa Maeno

**Affiliations:** Department of Virology and Parasitology, Fujita Health University School of Medicine, 1-98 Kutsukake, Toyoake, Aichi 470-1192 Japan

**Keywords:** Sporozoites, Gametocyte, Vietnam, *Anopheles dirus*, *Plasmodium vivax*, *Plasmodium falciparum*, *Plasmodium knowlesi*, *Plasmodium cynomolgi*, *Plasmodium coatneyi*, *Plasmodium inui*

## Abstract

Human infection caused by non-human primate malarial parasites, such as *Plasmodium knowlesi* and *Plasmodium cynomolgi*, occurs naturally in Southeast Asian countries, including Vietnam. Members of the *Anopheles dirus* species complex are known to be important vectors of human malarial parasites in the forested areas of southern and central Vietnam, including those in Khanh Phu commune and Khanh Hoa Province. Recent molecular epidemiological studies in Vietnam have reported cases of co-infection with *Plasmodium falciparum*, *Plasmodium vivax*, *Plasmodium malariae*, and *P. knowlesi* in *An. dirus*. The commonly found macaques in the forest in the forested areas are suspected to be bitten by the same *An. dirus* population that bites humans. A recent epidemiological study identified six species of malarial parasites in sporozoite-infected *An. dirus* using polymerase chain reaction, of which *P. vivax* was the most common, followed by *P. knowlesi*, *Plasmodium inui*, *P. cynomolgi*, *Plasmodium coatneyi*, and *P. falciparum*. Based on a gametocyte analysis, the same allelic gametocyte types were observed in both humans and mosquitoes at similar frequencies. These observations suggest that people who stay overnight in the forests are frequently infected with both human and non-human primate malarial parasites, leading to the emergence of novel zoonotic malaria. Moreover, it is suggested that mosquito vector populations should be controlled and monitored closely.

## Background

Malaria is among the most important infectious diseases caused by protozoans in humans. It is transmitted in tropical and subtropical areas. In 2015, about 212 million cases and 429,000 deaths related to Malaria were reported [1]. “Forest malaria” is a term frequently used to describe a malarial characteristic that is mainly found in Southeast Asia. Malarial parasite transmission occurs in the forested areas of the southern and central provinces of Vietnam [2, 3] and throughout other Southeast Asian countries. Malaria has become a public health concern in these areas.

Five species of malarial parasites, including *Plasmodium falciparum*, *Plasmodium vivax*, *Plasmodium malariae*, *Plasmodium ovale wallikeri*, and *Plasmodium ovale curtisi*, infect humans. On the other hand, 13 species of malarial parasites that infect non-human primates are found in Southeast Asian countries [4]. Among these non-human malarial parasites, *Plasmodium knowlesi* is now well known to infect humans and is considered a threat to human health in several Southeast Asian countries, including Vietnam [2, 5–8]. Recently, the first naturally acquired human *Plasmodium cynomolgi* infection was reported in the Malaysian peninsula [9]. Other malarial parasites that infect non-human primates, including *Plasmodium inui*, *Plasmodium eyesi*, and *Plasmodium schwetzi*, can infect humans through experimental or accidental infection [10–18]. These findings indicate that malarial parasites have the ability to switch hosts [19]. Malarial parasites that infect non-human primates can only cause zoonotic infection when mosquito vectors infected with these parasites encounter people. However, the process of zoonotic transmission is not sufficiently understood. Thus, malarial parasites in mosquitoes should be identified and characterized to fully understand the complexities of malarial parasite transmission.

The results of the epidemiological studies on malaria conducted in the Khanh Phu commune, Khanh Vinh, and Khanh Hoa Province, Vietnam, were used in this review. Khanh Phu is a commune with about 3000 residents, mainly of the Raglai ethnic minority. Most of the residents live between the forested foothills on the east side of the Truong Son mountain range in south-central Vietnam. Malaria in these areas was previously hyper- to holo-endemic.

### Anopheline species as a malaria vector

The characteristics of mosquito species can affect the transmission of malarial parasites between humans and non-human primates. Of about the 430 *Anopheles* species, only 25–30 are vectors of human and non-human primate malarial parasites [20, 21]. In the Greater Mekong Subregion, most often reported vectors were *Anopheles minimus*, *Anopheles dirus*, *Anopheles sundaicus*, *Anopheles sinensis*, and *Anopheles maculatus* [22]. In south and central Vietnam, the same *Anopheles* species that are considered as malaria vectors are collected [2, 3, 20, 22, 23]. However, the main vector in these areas is now *An. dirus* instead of *An. minimus*, *An. maculatus*, and other vectors [2, 3, 20].

### Biting rhythm of the *Anopheles* mosquito

**Fig. 1:**
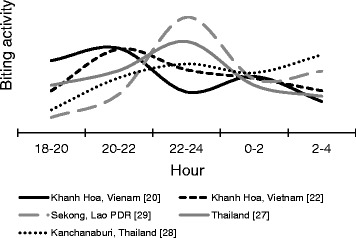
The biting rhythm of *Anopheles dirus* (*An. dirus*) outdoors. The hourly biting rhythm of *An. dirus* by collection sites (province and country) and reviewed publication [reference]. Presented data in this figure was converted to hourly percentages to allow comparison of data from various sources

In Khanh Phu commune, *An. dirus* mosquitoes were collected every month with an average human-biting density of 3.5 per person per night (Table 1) [20, 24, 25]. As shown in Fig. 1, *An. dirus* started to bite humans immediately after sunset, which peaks at 20:00–22:00 and then 0:00–2:00 [20]. A similar biting rhythm of *An. dirus* was found in the previous report in Vietnam. However, a late biting activity of *An. dirus* was observed in Thailand and Lao PDR [22, 26–29]. In relation to biting activity and environmental factors, biting density in the forest is higher than that in the village [26]. This biting density did not differ between forest fringe and forest [20]. The early biting activity of *An. dirus* is dependent on species, location, and other factors. Previously, nocturnal rainfall did not affect the biting activity of this species in the Khanh Phu commune, and no difference in the biting activity was observed between rainy and dry nights [22]. The biting activity rate was increased in moonlight nights than moonless nights [22] and in the outdoors than the indoors [26, 30]. In areas including the Khanh Hoa Province and Ninh Thuan Province, residents often sleep outdoors in hammocks or plot huts in the forests. This activity causes humans to be more susceptible to mosquito bites. These findings suggest that the transmission of malarial parasites occurs because of a dependent pattern of behavior between humans and mosquitoes.

**Table 1:** Results of the collection, dissections, and PCR processing of *Anopheles dirus* (*An. dirus*) in the forest of study area [20, 24, 25]

Year	Nights	No. caught	Biting density^a^	No. dissection	No. sporozoites	Percent
2008	724	2120	2.93	2119	22	1.04
2009	676	2856	4.22	2848	32	1.12
2010	674	3218	4.77	3207	77	2.40
2011	659	1601	2.43	1594	21	1.32
To Oct. 2012	563	1669	2.96	1669	16	0.96
Total	3296	11,464	3.48	11,437	168	1.47

^a^Biting density, average human-biting density (No. of caught/No. of caught person per night)

### Prevalence of malaria infection in mosquito species

Recent molecular epidemiological studies in Vietnam reported the co-infection of *P. falciparum*, *P. vivax*, *P. malariae*, and *P. knowlesi* in *An. dirus* [2, 31]. The commonly found macaques in the forests of the study area are suspected to be bitten by the same *An. dirus* population that bites humans. To resolve the problem, a total of 120 female *An. dirus* mosquitoes infected with sporozoites, collected at the forest and forest fringe in the Khanh Phu commune, were analyzed for the detection of malarial parasite species using nested PCR (Table 2). Based on the analysis, six species of malarial parasites were detected, of which *P. vivax* is the most common and followed by *P. knowlesi*, *P. falciparum*, *P. inui*, *P. cynomolgi*, and *Plasmodium coatneyi*. However, *P. malariae* and *Plasmodium ovale* were not detected among the analyzed mosquito samples (Table 2).

**Table 2:** Summary of *Plasmodium* spp. infections in *Anopheles dirus* [20, 24, 25]

Infection	*Plasmodium* spp.	No. of mosquitoes infected
Single (71)	Pf	15
	Pv	34
	Pk	4
	Pct	7
	Pcy	6
	Pin	5
Double (30)	Pf, Pv	5
	Pf, Pin	1
	Pv, Pk	15
	Pv, Pcy	3
	Pv, Pin	3
	Pk, Pin	3
Triple (13)	Pf, Pv, Pk	6
	Pf, Pv, Pin	1
	Pv, Pk, Pcy	1
	Pv, Pk, Pin	2
	Pv, Pct, Pin	1
	Pk, Pct, Pin	2
Quadruple	Pv, Pct, Pcy, Pin	1
No. of PCR-positive		115
No. of PCR-negative		5
Total no. of examined sporozoite-positive		120

*Pf P. falciparum*, *Pv P. vivax*, *Pk P. knowlesi*, *Pct P. coatneyi*, *Pcy P. cynomolgi*, *Pin P. inui*

Those sporozoite-positive *An. dirus* showed single and mixed species parasite infection. Among the vectors that cause single species infection, *P. vivax* was dominant, followed by *P. falciparum*, *P. coatneyi*, *P. cynomolgi*, *P. inui*, and *P. knowlesi* (Table 2). On the other hand, the co-infections of malarial parasite species were common, with 38% of mosquitoes infected by two or more species of the malarial parasites, with most cases considered as mixed species infection caused by *P. vivax* and another species (Table 2). As shown in Table 2, *P. vivax* co-infections with non-human primate malaria species, such as *P. knowlesi*, *P. inui*, *P. cynomolgi*, and *P. coatneyi*, were observed. For *P. falciparum*, co-infection with non-human primate *Plasmodium* species was observed only in *P. inui*. In contrast, co-infection among human *Plasmodium* species, such as *P. vivax* and *P. falciparum* (including six cases of co-infection with *P. vivax* and *P. knowlesi*), was observed. Similar molecular epidemiological studies using mosquitoes as a target are not available. However, Lee et al. [32] reported that nested PCR analysis of blood samples of wild macaques, which were collected in the Kapit Division of Sarawak, Malaysia, detected five non-human primate malarial parasites, including *P. inui*, *P. knowlesi*, *P. coatneyi*, *P. cynomolgi*, and the *Plasmodium fieldi*. Among them, single and mixed species infections were observed. These findings from mosquitoes and macaques indicated that transmission of malarial parasites in mosquitoes is dependent on the monkey reservoir and/or was maintained between humans. Therefore, further study should be conducted to obtain detailed information about the transmission of malarial parasites.

The data show that *An. dirus* is infected with two species of human malarial parasites (*P. falciparum* and *P. vivax*) and four species of non-human primate malarial parasites (*P. knowlesi*, *P. inui*, *P. cynomolgi*, and *P. coatneyi*) (Table 2). These results suggest that both humans and macaques are bitten by *An. dirus* in the forests. To obtain data and verify whether and which macaques are the reservoirs of non-human primate malarial parasites, macaque fecal samples that were collected from forest floors and a cage in the forest where *An. dirus* is commonly found were analyzed. Among these macaques, the caged macaques were infected with *P. cynomolgi*, *P. coatneyi*, *P. inui*, and *P. knowlesi*, of which the latter species is only found in fecal samples [33]. As shown in Table 2, approximately one-third of *An. dirus* were infected by two or more species of malarial parasites, and these were often a combination of human and non-human primate parasites. The numerous co-infections and the lack of differences between the collection sites and biting times further suggest that all these parasites are transmitted by only one population of *An. dirus*, which bites humans as readily as they bite macaques.

### The emergence of novel zoonotic malarial infections

*P. knowlesi* is considered as the “fifth human malarial parasite,” which is somehow controversial. The controversy stems from the fact that whether this parasite can be transmitted from human to human is not known. If human infection only resulted from a “spill-over” of transmission between monkeys, then the infection must be considered a zoonotic disease rather than a human one. First, to determine whether *P. knowlesi* is also infectious to mosquitoes, its capability of producing gametocytes should be investigated. Previous studies have reported the presence of *P. knowlesi* gametocytes, identified by microscopy, in the blood of infected patients [5, 34–36]. Recently, transcripts of *pks25*, the *P. knowlesi* orthologue of *Pvs25* [37], in dried blood samples of people infected with *P. knowlesi* in the Khanh Phu commune was detected, although those samples did not show either gametocytes or the asexual forms of *P. knowlesi* through a microscopy [25]. This finding indicates that *P. knowlesi*, which infects humans in the Khanh Phu commune, may also be infectious to mosquitoes.

Both the behavior of humans and recent findings on transmission indicate that humans who stay overnight in forests are frequently infected with a range of similar malarial parasites, several of which have been shown to cause diseases in humans [2, 5–10, 13, 16, 17, 38]. The implications of this are potentially serious. Given that malarial parasites are capable of infecting different species and causing zoonotic infections under certain circumstances, exposure is the major factor that causes new zoonotic infection. Humans who are routinely and regularly exposed to inoculations of (currently) non-human primate sporozoites are at high risk for novel zoonotic malarial infections.

## Conclusion

Recent studies in the Khanh Phu commune indicated that both human and non-human primate malarial parasite sporozoites from the same mosquito population were considered as a characteristic of malaria, and a similar frequency of the same allelic types of gametocytes was also observed in humans and mosquitoes captured in the forest area. These findings suggest that humans who stay overnight in the forests are frequently infected with both human and non-human primate malarial parasites, thus causing the emergence of novel zoonotic malaria. Moreover, these findings suggest that mosquito vector populations should be controlled and monitored closely.
